# Supplementary material for: Visible Photocatalytic Hydrogen Evolution by g-C3N4/SrZrO3 Heterostructure Material
Source: Nanomaterials (Basel). 2023 Mar 8;13(6):977. doi: 10.3390/nano13060977 (PMC10057068; doi:10.3390/nano13060977)
Supplement: Supplementary file 1 [file nanomaterials-13-00977-s001.zip › nanomaterials-2231122-supplementary.pdf]

nanomaterials

MDPI

Supplementary Material

Visible Photocatalytic Hydrogen Evolution by g-C_3N_4/$SrZrO_3$ Heterostructure Material

Shizhao Si †, Yanfei Fan †, Dan Liang, Ping Chen, Guanwei Cui * and Bo Tang

College of Chemistry, Chemical Engineering and Materials Science, Collaborative Innovation Center of Functionalized Probes for Chemical Imaging in Universities of Shandong, Key Laboratory of Molecular and Nano Probes, Ministry of Education, Shandong Normal University, Jinan 250014, China

* Correspondence: cuiguanwei@sdnu.edu.cn

† These authors have contributed equally to this work and share first authorship.

1. Supplementary Figures

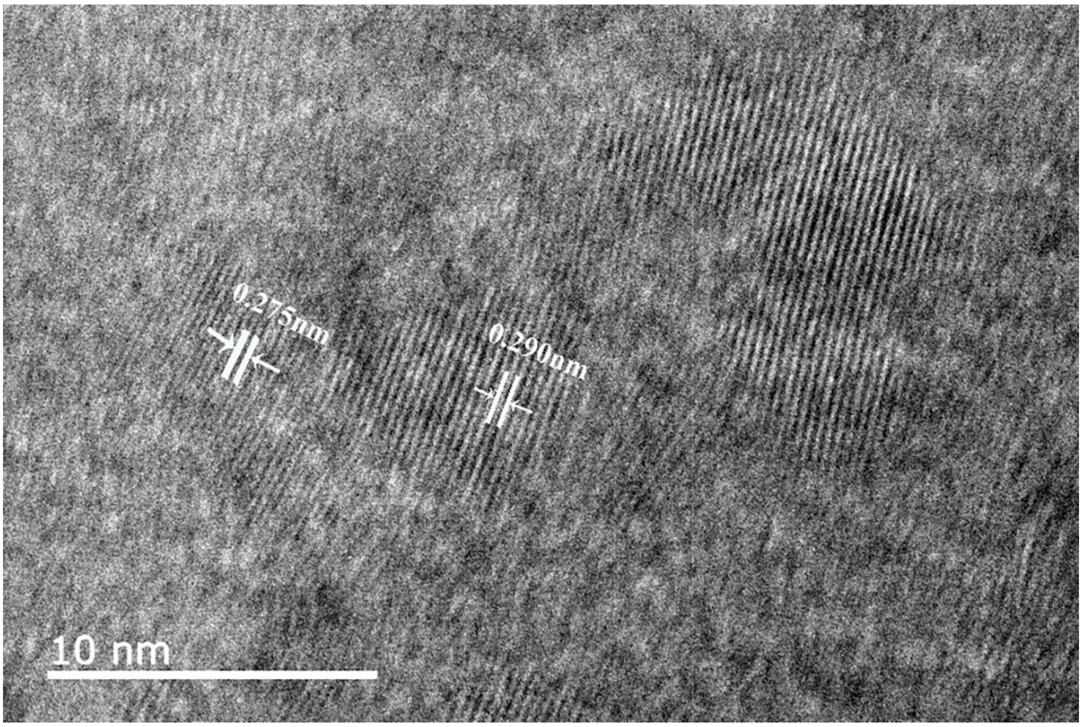

Figure S1 HRTEM image of g-C_3N_4/$SrZrO_3$

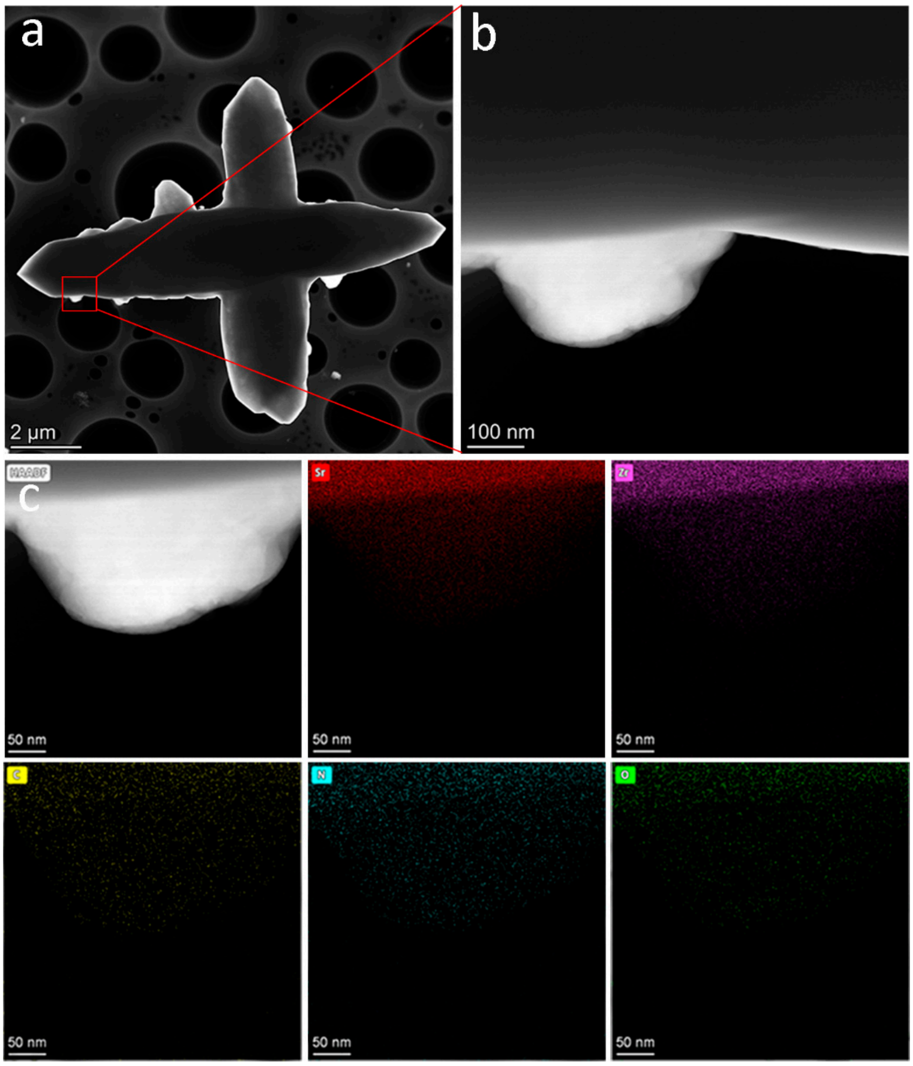

Figure S2 The images of g-C3N4/SrZrO3 HAADF-TEM (**a**)(**b**) and corresponding EDS mapping (**c**).

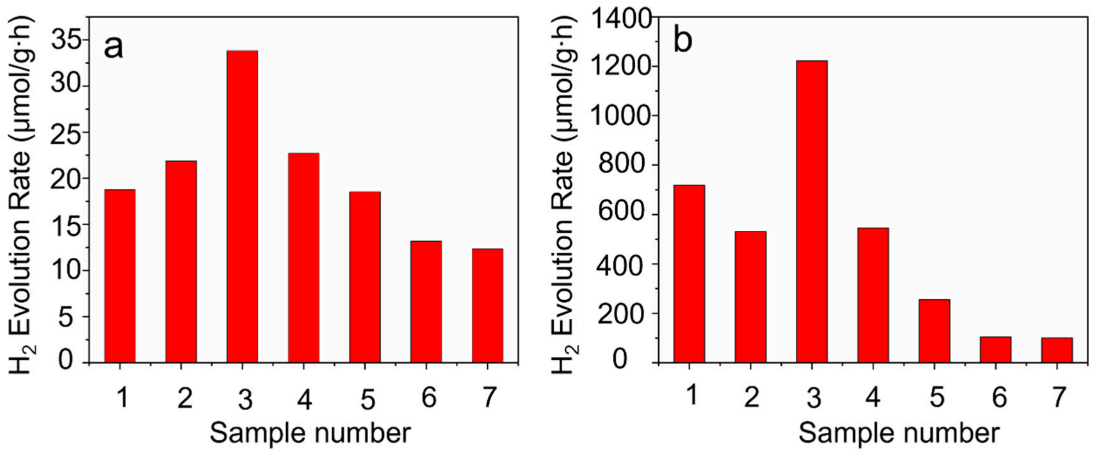

Figure S3 Hydrogen evolution under visible light (**a**) and UV light (**b**) for different ratios of g-C_3N_4/$SrZrO_3$ catalysts

Sample numbers 1-7 are g-C_3N_4/$SrZrO_3$ catalysts in the ratios of 1:20, 1:15, 1:10, 1:5, 1:3, 1:1 and 2:1 for g-C_3N_4/$SrZrO_3$.

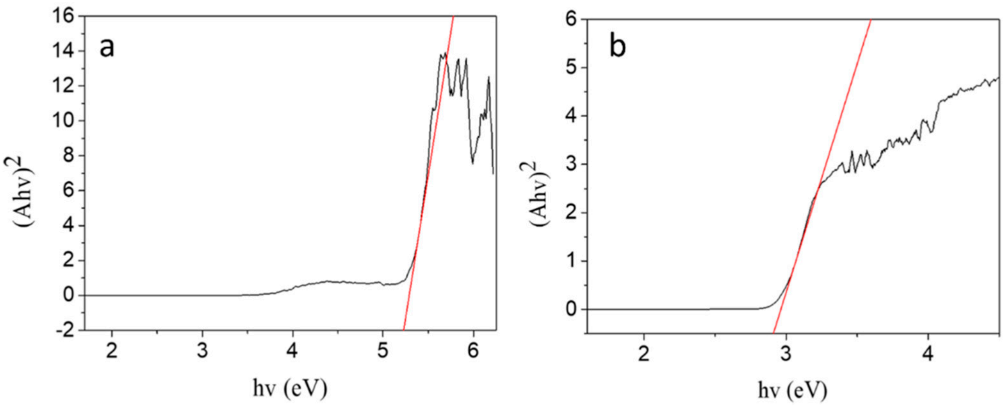

Figure S4 Energy gap of $SrZrO_3$ (**a**) and g-C_3N_4 (**b**)

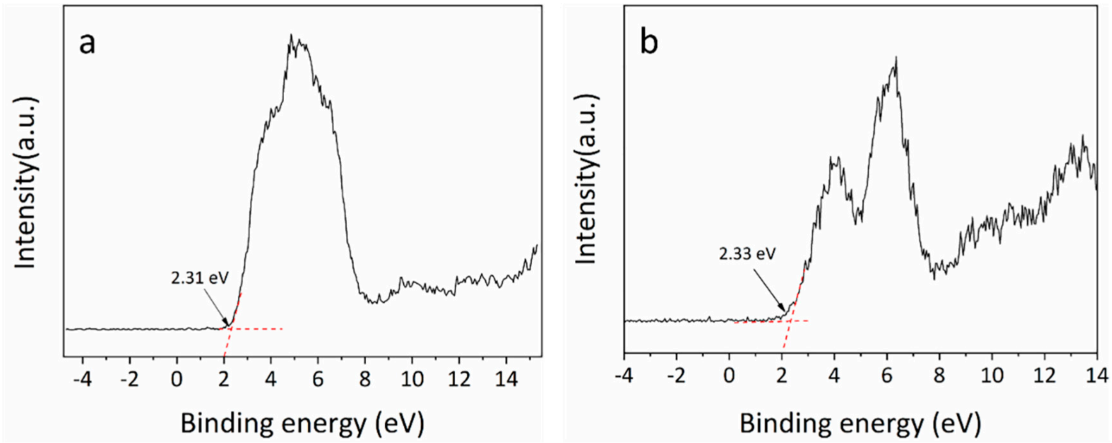

Figure S5 XPS valence spectrum of $SrZrO_3$(**a**), g-C_3N_4(**b**)

Table S1. The selected results for photocatalytic H_2 evolution in recent literature.

Catalyst	Scavenger	Light source	H_2 yield (μmol $g_{cat}^{-1}h^{-1}$)	Reference
$SrZrO_3$-MOF (LEEL-037)	No	UV light	66.9	[1]
$SrZrO_3$-Sb_2O_3	No	UV light	330	[2]
$SrZrO_3$-CuO	No	UV light	1165	[3]
Pd-CdS/g-C_3N_4	Na_2S and Na_2SO_3	Full spectrum	293	[4]
g-C_3N_4/$SrZrO_3$	Methanol	UV light	1222	This work

References

1. Alfonso-Herrera, L.A.;Huerta-Flores, A.M.; Torres-Martínez, L.M. Hybrid $SrZrO_3$-MOF heterostructure: surface assembly and photocatalytic performance for hydrogen evolution and degradation of indigo carmine dye. *J. Mater. Sci. Mater. Electron.* **2018**, *29*, 10395–10410.
2. Huerta-Flores, A.M.; Torres-Martínez, L.M.; Moctezuma, E. Novel $SrZrO_3$-Sb_2O_3 heterostructure with enhanced photocatalytic activity: Band engineering and charge transference mechanism. *J. Photochem. Photobiol. A*. **2018**, *356*, 166–176.
3. Huerta-Flores, A.M.; Torres-Martínez, L.M.; Moctezuma, E. Enhanced photocatalytic activity for hydrogen evolution of $SrZrO_3$ modified with earth abundant metal oxides (MO, M = Cu, Ni, Fe, Co). *Fuel*. **2016,** 181, 670–679.
4. Güy, N. Directional transfer of photocarriers on CdS/g-C_3N_4 heterojunction modified with Pd as a cocatalyst for synergistically enhanced photocatalytic hydrogen production. *Appl. Surf. Sci*. **2020**, *522*, 146442.
